# P-2326. The Under-recognized Burden of Lassa Virus Infection in Liberia

**DOI:** 10.1093/ofid/ofae631.2478

**Published:** 2025-01-29

**Authors:** Catherine Nimely, Emmanuel Kerkula, Taylor Krajewski, McKenzie Colt, Alfred Flomo, Amara Fofana, Stanley Kerkula, Thomas Remont, Rosie Watts, Sam Tozay, Katie R Mollan, Paul Zivich, Minnie Ricks, David A Wohl, William A Fischer, Jefferson Sibley

**Affiliations:** UNC Global Projects-Liberia, Bong, Bong, Liberia; UNC-Liberia, Bong, Bong, Liberia; UNC Gillings School of Public Health, Chapel Hill, North Carolina; UNC-Liberia, Bong, Bong, Liberia; UNC-Liberia, Bong, Bong, Liberia; UNC-Liberia, Bong, Bong, Liberia; UNC-Liberia, Bong, Bong, Liberia; UNC-Liberia, Bong, Bong, Liberia; UKHSA, Andover, England, United Kingdom; Gillings School of Public Health, Chapel Hill, North Carolina; UNC Gillings School of Public Health, Chapel Hill, North Carolina; Gillings School of Public Health, Chapel Hill, North Carolina; Phebe Hospital, Bong, Bong, Liberia; University of North Carolina at Chapel Hill School of Medicine, Chapel Hill, North Carolina; Institute for Global Health and Infectious Diseases, Chapel Hill, North Carolina; Phebe Hospital, Bong, Bong, Liberia

## Abstract

**Background:**

Lassa virus (LASV) is a persistent and lethal threat in endemic regions of West Africa. While severe disease due to Lassa fever (LF) is responsible for 5000-10,000 deaths annually, there is evidence of a wide spectrum of clinical manifestation of LASV infection.

**Methods:**

The Coalition for Epidemic Initiative (CEPI) sponsored ENABLE study is a prospective, observational, cohort study of the prevalence and incidence of LASV infection and LF in five West African countries: Nigeria, Liberia, Sierra Leone, Guinea, and Benin. LASV seroprevalence was determined by LASV nucleoprotein (NP) IgG at entry (ReLASV NP IgG ELISA). Participants were assessed every 2 weeks in-person for fever with and without LF symptoms. Febrile events triggered LASV RT-PCR (RealStar Lassa Virus RT-PCR Altona 2.0). To assess for subclinical LASV infection, serum was collected every 6 months for 2 years from a subset (Infection Cohort) for LASV NP IgG.

**Results:**

In Liberia, 5,005 were enrolled between April 2021 and December 2023; 25 withdrew before data collection. Of the remaining 4,980 participants, 1,018 were in the Infection Cohort and 591 of these provided samples at all 5 time points. At entry, 2,259 (45%) were LASV NP IgG positive. Entry LASV NP IgG seropositivity increased with age with the lowest prevalence among those 2-5 years (101 of 510, 20%, CI: 17-24%) rising to a peak among participants 30-39 years (322 of 564, 57%, CI: 53-61%). Of the 591 with serological results at all 5 timepoints, 314 (53%) were LASV NP IgG negative at study entry and 68 (22%) had at least a single change in serostatus from seronegative to seropositive. Over 24-months, 1,176 febrile events in 818 participants were identified and of these 10 (1%) had LASV RNA detected.

**Conclusion:**

In this 24-month longitudinal study of individuals in a LASV hyperendemic area of West Africa, close to half were LASV seropositive at enrollment, and across two years of follow-up, 1 in 5 participants who were LASV seronegative at entry had observed seroconversion. However, only a small number of LF cases (n=14) were identified through symptom-driven testing dependent on fever. These findings indicate that in this region of Liberia LASV infection is common, increases during the first decades of life, but is rarely clinically detected.

**Disclosures:**

Katie R. Mollan, MS, Gilead Sciences: Grant/Research Support|Ridgeback Biotherapeutics: Grant/Research Support

